# Camonsertib, an ATRi, in Combination with Low-Dose Gemcitabine in Solid Tumors with DNA Damage Response Aberrations: Preclinical and Phase Ib Results

**DOI:** 10.1158/1078-0432.CCR-25-2240

**Published:** 2026-01-21

**Authors:** Ezra Y. Rosen, Timothy A. Yap, Elisa Fontana, Elizabeth K. Lee, Devalingam Mahalingam, Martin Højgaard, Niharika B. Mettu, Gregory M. Cote, Ruth Plummer, Maria Koehler, Danielle Ulanet, Kezhen Fei, Ian M. Silverman, Joseph D. Schonhoft, Victoria Rimkunas, Emeline S. Bacque, Gabriela Gomez, Adrian J. Fretland, Anne Roulston, Li Li, Prasamit Baruah, Michal Zimmermann, Julia Yang, Benedito A. Carneiro, Stephanie Lheureux

**Affiliations:** 1 Early Drug Development and Breast Medicine Services, Division of Solid Tumor Oncology, Department of Medicine, Memorial Sloan Kettering Cancer Center, New York, New York.; 2 Investigational Cancer Therapeutics, The University of Texas MD Anderson Cancer Center, Houston, Texas.; 3 Sarah Cannon Research Institute UK, London, United Kingdom.; 4 Medical Oncology, Dana-Farber Cancer Institute, Boston, Massachusetts.; 5 Robert H. Lurie Comprehensive Cancer Center, Division of Hematology/Oncology, Northwestern University Feinberg School of Medicine, Chicago, Illinois.; 6 Department of Oncology, Rigshospitalet, Copenhagen, Denmark.; 7 Medical Oncology, Duke University, Durham, North Carolina.; 8 Massachusetts General Hospital Cancer Center, Boston, Massachusetts.; 9 Sir Bobby Robson Cancer Trials Research Centre, Freeman Hospital, Newcastle upon Tyne, United Kingdom.; 10 Repare Therapeutics, Cambridge, Massachusetts.; 11 Repare Therapeutics, St-Laurent, Canada.; 12 Legorreta Cancer Center at Brown University and Lifespan Cancer Institute, Division of Hematology/Oncology, Department of Medicine, The Warren Alpert Medical School, Brown University, Providence, Rhode Island.; 13 Princess Margaret Cancer Centre, Toronto, Canada.

## Abstract

**Purpose:**

The utility of combination treatment with gemcitabine and camonsertib, an ataxia telangiectasia and Rad3-related kinase inhibitor, in mediating tumor cell death was assessed in preclinical models, prompting clinical investigation. The phase Ib TRESR study (NCT04497116) aimed to evaluate the safety, tolerability, and preliminary efficacy of the combination in patients with advanced solid tumors harboring DNA damage repair (DDR) gene alterations.

**Patients and Methods:**

Cell lines and tumor xenografts were tested across a range of dose levels and schedules. Patients (*N* = 76) harboring tumors with DDR gene alterations received camonsertib (80–120 mg) and de-escalating gemcitabine (1,000–100 mg/m^2^) in 21- or 28-day cycles on an intermittent dosing regimen. Safety, tolerability, and preliminary efficacy were assessed to identify an optimal dosing regimen.

**Results:**

In preclinical models, low-dose camonsertib (1/3 maximum tolerated dose) and gemcitabine led to tumor regression and was well tolerated with minimal body weight loss observed. In patients, synergistic toxicities were observed, primarily myelosuppression, resulting in gemcitabine de-escalation. The introduction of a 1 week on/1 week off schedule in combination with low-dose gemcitabine allowed for spontaneous neutrophil recovery, fewer dose modifications, and improved tolerability. Tumor responses were primarily observed in patients with gynecologic cancers, with tumor control maintained for greater than 1 year in some patients.

**Conclusions:**

Camonsertib and low-dose gemcitabine demonstrated preliminary clinical activity, but due to challenging tolerability, further evaluation is warranted to identify the optimal dosing regimen and subset of patients who may benefit most from this combination.

**Translational Relevance**

The work presented here details the safety, tolerability, and preliminary efficacy data of combined gemcitabine and the ataxia telangiectasia and Rad3-related kinase inhibitor (ATRi) camonsertib. The mechanism of action for both gemcitabine and camonsertib is mediated through replication stress response pathways, thereby sensitizing tumors dependent on ATR. However, both drugs are potentially toxic and myelosuppressive, prompting evaluation of low-dose gemcitabine and intermittent dosing schedules. In this study, we demonstrated proof of concept that camonsertib can be combined with low-dose gemcitabine on a 1 week on/1 week off schedule to achieve antitumor activity with manageable tolerability in heavily pretreated patients harboring tumors with a loss-of-function gene alteration in DNA damage repair genes predicted to be synthetic lethal with ATRi.

## Introduction

Inhibition of the ataxia telangiectasia and Rad3-related kinase (ATR) has emerged as a potential therapeutic strategy for treating cancer with selected molecular defects in DNA damage response (DDR) and repair pathways (1–4). ATR itself plays a crucial role in DDR by regulating cell-cycle checkpoints and DNA repair under conditions of DNA replication stress. Upon activation by single-stranded DNA breaks, ATR coordinates multiple key activities, including inhibition of cell-cycle progression, regulation of DNA origin firing, replication fork reversal, and homologous recombination (HR; ref. 5). Camonsertib is a highly potent and selective oral inhibitor of ATR kinase (6), with demonstrated activity in patients harboring tumors with loss-of-function (LoF) alterations in *ATM*, *BRCA*1/2, and other DDR genes (3, 7) and a manageable safety profile, consistent with other well-characterized DDR inhibitors (8). The central role of ATR in the physiologic response to replication stress provides a compelling rationale for combining an ATR inhibitor (ATRi) with replication stress–inducing antitumor agents (1). Gemcitabine (2′2′-difluorodeoxycytidine) is a nucleoside analog antimetabolite that induces profound replication stress by inhibiting ribonucleotide reductase and by directly incorporating into DNA (9). As such, gemcitabine treatment increases the cellular dependence on ATR, and early preclinical studies demonstrate a profound potentiation of tumor cytotoxicity of ATRi when combined with gemcitabine (10–13). To date, ATRi plus gemcitabine combinations have been evaluated in three phase I/II clinical trials utilizing a first-generation ATRi, berzosertib (14–16). These initial studies highlighted the potential of this drug combination to improve outcomes versus gemcitabine alone. In an effort to maximize the clinical activity of camonsertib, we performed preclinical proof-of-concept studies which supported a phase Ib clinical study to evaluate the safety, tolerability, and efficacy of the combination of camonsertib and low-dose gemcitabine.

## Patients and Methods

### Cell lines and cell culture

SUM149PT cells were purchased from Applied Biological Materials (RRID: CVCL_3422) and cultured in Ham’s F12 medium supplemented with 5% FBS (VWR, 76419-584), 1 µg/mL hydrocortisone (STEMCELL Technologies, 07925), 5 µg/mL insulin (MilliporeSigma, I1882-100MG), 100 U/mL penicillin (pen), and 100 μg/mL streptomycin (pen/strep; Corning, 30-001-CI). RPE1 (RRID: CVCL_4388)*TP53-*knockout (*KO*) *ATM–*wild-type (*WT*) and *ATM-KO* cells were described previously (6) and grown in DMEM supplemented with 10% FBS and pen/strep. Granta-519 cells (RRID: CVCL_1818; DSMZ) were cultured in DMEM with 4.5g/L glucose and sodium pyruvate, supplemented with 2 mmol/L L-glutamine, 10% heat-inactivated FBS, and pen/strep as above. All cell lines were maintained at 37°C with 5% CO_2_ (10% CO_2_ for Granta-519) and tested for *Mycoplasma* contamination.

### Cell viability assays

Camonsertib was synthesized by Omega Chemicals. Gemcitabine was purchased from MedChemExpress. Stock solutions of compounds were made up in DMSO to a concentration of 10 mmol/L and kept at −20°C for long-term storage.

On day 0, cells were seeded on white, clear bottom 96-well plates (Corning, 3903), and the test compounds were added on day 1 using an automated Tecan D300E dispenser. Where indicated, compound-containing media were removed upon an incubation period and replaced with drug-free media. Cells were grown until untreated wells reached near-confluence (as indicated in respective figures), after which cell viability was measured using a CellTiter-Glo assay kit (Promega) according to the manufacturer’s instructions. ZIP scores between camonsertib and gemcitabine were using the ZIP model in SynergyFinder v3.0 (RRID: SCR_019318; refs. 17, 18).

### *In vivo* xenograft studies

*In vivo* studies using the Granta-519 model were conducted at Crown Biosciences, Inc. in accordance with the regulations of the Association for Assessment and Accreditation of Laboratory Animal Care with a protocol approved by Crown’s Institutional Animal Care and Use Committee. *In vivo* studies using SUM149PT cells were conducted at the Repare Therapeutics (NEOMED site), which is a Canadian Council on Animal Care–accredited vivarium. Studies were conducted under a protocol approved by the NEOMED Institutional Animal Care Committee. Tumor cells (Granta-519 at1 × 10^7^ cells/mouse and SUM149PT at 5 × 10^6^ cells/mouse) were subcutaneously implanted into the flanks of female NOD/SCID mice (Granta-519: RRID: IMSR_JAX:001303 and SUM149PT: Charles River Laboratories) in 1:1 Matrigel:PBS. Once tumors reached an average 100 to 150 mm^3^ in volume, mice were randomized to groups, and treatment was initiated simultaneously for all groups. Mice were monitored for tumor volume (TV), clinical signs, and body weight 3 times per week. TV was measured by vernier caliper and calculated using the formula TV = 0.52 × L × W^2^. Camonsertib was administered per-oral once daily on an intermittent schedule of 3 days on/4 days off (3/4d) weekly in 0.5% methylcellulose/0.02% SDS vehicle. Gemcitabine was administered intraperitoneally once weekly in PBS pH 7.4. At experiment termination, blood was collected by cardiac puncture into precoated ethylenediaminetetraacetic acid tubes and analyzed on a hematology analyzer (Sysmex Corporation). Tumor growth inhibition (TGI) was defined as TGI = [(TV_vehicle/last_ − TV_vehicle/day0_) − (TV_treated/last_ − TV_treated/day0_)]/(TV_vehicle/last_ − TV_vehicle/day0_) × 100% calculated based on the means of the treatment groups at day 0 and last day of measurement. TV is tumor volume, and subscripts indicate treatment group and time of sampling.

### Study design and patients

The TRESR phase I/IIa study (NCT04497116) enrolled 76 patients with molecularly selected advanced solid tumors treated with the combination of camonsertib and gemcitabine as a subpart of this protocol. As of the data cutoff on December 11, 2024, all patients had at least 10 months follow-up or discontinued treatment prior to 10 months. Key inclusion criteria included age of ≥18 years at the time of consent, Eastern Cooperative Oncology Group Performance Status score 0 or 1, baseline hemoglobin ≥10 g/dL, histologically confirmed solid tumors resistant or refractory to standard treatment, and/or patients intolerant to standard therapy. To meet molecular eligibility, a deleterious, or likely deleterious, alteration in at least one of the following genes was required: *ATM*, *ATRIP*, *BRCA1*, *BRCA2*, *CDK12*, *CHTF8*, *FZR1*, *MRE11*, *NBN*, *PALB2*, *RAD17*, *RAD50*, *RAD51B/C/D*, *REV3L*, *RNASEH2A*, *RNASEH2B*, *SETD2*, or other genes agreed with the sponsor and investigator. Genetic eligibility was centrally confirmed and annotated by the University of Texas MD Anderson Cancer Center Precision Oncology Decision Support Group. Key exclusion criteria included treatment with chemotherapy, small molecules, or biologic anticancer therapy within 14 days prior to first dose of the study drug, prior therapy with an ATR or DNA-dependent protein kinase inhibitor, or prior therapy with a gemcitabine-containing regimen as the most recent prior line of therapy (prior gemcitabine is allowed if there is at least one intervening therapy). Patients were enrolled initially into dose-escalation cohorts, with Safety Review Committee (SRC) approval required prior to opening of new dose levels. Expansion cohorts were opened with SRC approval to further assess possible antitumor activity and drug combination–related toxicities in a subset of patients with a specific genomic abnormality or tumor type.

The study was conducted in accordance with the Declaration of Helsinki and Council for International Organizations of Medical Sciences International Ethical Guidelines, applicable International Conference on Harmonization, Good Clinical Practice Guidelines, and applicable laws and regulations. All patients provided written informed consent to adhere to the clinical protocol and provided serial blood samples. The protocol was approved by the Institutional Review Board or Ethics Committee at each participating institution listed in Supplementary Table S1.

### Objectives/endpoints

The primary objective of this analysis was to assess safety and tolerability, define the maximum-tolerable dose (MTD), characterize the recommended phase II dose (RP2D), and determine the schedule of camonsertib and gemcitabine combination treatment. The secondary objectives were to assess preliminary antitumor activity of the combination and the pharmacokinetics (PK) of camonsertib when combined with gemcitabine.

Endpoints for long-term safety/tolerability included treatment-emergent adverse events (TEAE), serious adverse events, and dose modifications due to treatment-related adverse events (TRAE). Efficacy endpoints included overall response rate (ORR) per RECIST or tumor marker response (TMR), cancer antigen-125 [CA-125; per Gynecological Cancer InterGroup (GCIG) or PSA per Prostate Cancer Clinical Trials Working Group 3], clinical benefit rate [CBR; RECIST or TMR response or treatment duration of at least 16 weeks (w) without evidence of progression], and progression-free survival (PFS). Camonsertib PK were assessed using liquid chromatography with tandem mass spectrometry analysis of plasma camonsertib concentrations at defined time points; PK parameters for camonsertib were determined using noncompartmental analysis using Phoenix version 8.3.3.33 (Certara; RRID: SCR_021370) and included AUC from time 0 to last quantifiable concentration (AUC_0–last_), AUC from 0 to 4  hours (h) after dose (AUC_0–4_), maximum observed plasma concentration (C_max_), as well as dose normalized C_max_, AUC_0–last_, and AUC_0–4_.

### Statistical analysis

The planned total number of patients exposed in module 4 was 70, excluding an estimated 10% to 15% that may be nonevaluable for dose-limiting toxicity (DLT). This number was deemed sufficient to enable a good understanding of drug-related toxicity. Dose-escalation decisions were governed by the BOIN design (19) and confirmed at the SRC meetings at the end of the DLT observation period.

Baseline demographics, disease characteristics, and TEAEs were summarized with descriptive statistics by dose levels or by schedule within the safety population. AEs were defined by Medical Dictionary of Regulatory Activities v27.0 (RRID: SCR_003751). The DLT rate was based on the DLT-evaluable population, which included those patients who received ≥80% of planned total doses of camonsertib and 100% of gemcitabine, who completed all required safety evaluations and were observed through the end of cycle 1, or patients who experienced a DLT. Efficacy endpoints were summarized among patients with ≥1 post-baseline tumor assessment or sufficient post-baseline tumor marker assessments (efficacy population). Additional subgroup efficacy analysis was done by dose level and among patients with and without gynecologic malignancies (patients with cervical, endometrial, ovarian, and uterine tumors) out of the efficacy population. No formal statistical tests were performed on primary or secondary endpoints, including baseline demographics (i.e., age, sex, and race/ethnicity) which were collected by the investigator and were generally comparable with phase I clinical trial participants in the United States (Supplementary Table S2).

### Retrospective clinical genomic analysis

Blood was collected pre-treatment and on day 1 (D1) of each cycle. Cell-free DNA was isolated and sequenced as previously described, using the commercially available Guardant360^(R)^ assay with 750-gene panel for genomic variant detection and Guardant Reveal™ assay for methylation-based ctDNA detection and quantification of circulating tumor fraction (cTF), both supported by Guardant Infinity™ next-generation sequencing (NGS) technology. Molecular response (MR) was defined in this study as the best response of ≥50% reduction in circulating TF (cTF) from baseline.

The enrollment LoF alteration was confirmed retrospectively by sequencing archival tissue using a custom next-generation sequencing (NGS) panel (SNiPDx; Repare Therapeutics, Inc) as previously described (3, 21).

## Results

### Preclinical studies evaluating the combination of camonsertib and low doses of gemcitabine

**Figure 1.**
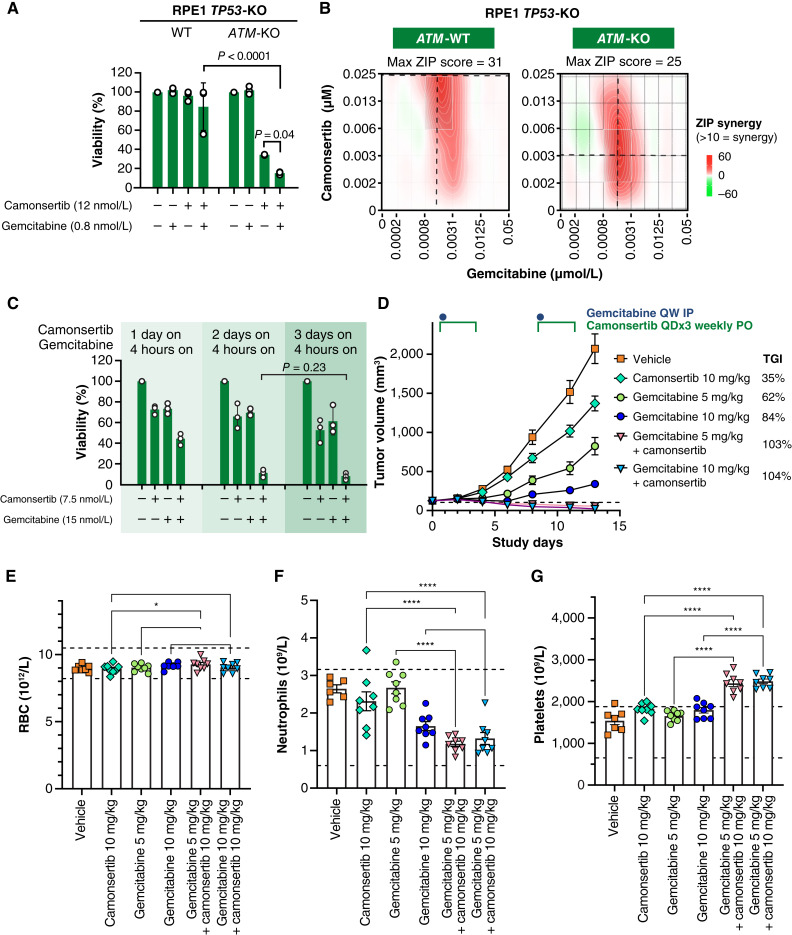
Preclinical data: combination of low doses of camonsertib and gemcitabine. **A,** Cell viability (as measured by CTG) of RPE1 *TP53*-KO *ATM*-WT or *ATM*-KO cells after indicated treatments. **B, ***In vitro*, low doses of camonsertib and gemcitabine elicit sensitivity in ATM-deficient cells. ZIP combination scores in RPE1 *TP53*-KO *ATM*-WT or *ATM*-KO cells treated with indicated concentrations of cam and gemcitabine and processed for a CTG viability assay are shown. **C, ***In vitro*, low doses of camonsertib and gemcitabine elicit cytotoxicity in *BRCA1*-deficient cells. Viability of SUM149PT cells (*BRCA1*-mutant triple-negative breast carcinoma) as measured by a CTG growth assay. Cells were either left untreated or treated with 4 hours of gemcitabine, day 1, 2, or 3 of camonsertib, or combined camonsertib and gemcitabine. Compounds were then washed out, and viability was measured on day 8. **D,** Mice bearing Granta-519 tumors were treated with camonsertib (3/4 days weekly) or gemcitabine (once weekly) as single agents and in combination at the indicated doses. Tumor growth is represented as the mean ± SEM; *n* = 8 mice/group. Statistical differences between groups were determined using an unpaired *t* test with Welch correction. ***, *P* < 0.001; ****, *P* < 0.0001 (Prism v10.4). At termination of the experiment, complete blood counts were taken from all mice. **E,** Mean RBC, **F,** neutrophils, and **G,** platelets are shown ±SEM. Dashed lines represent normal range for age-, sex-, and strain-matched mice. Statistical differences were determined using one way ANOVA and Fisher LSD test (Prism v10.4). ns, not significant; *, *P* < 0.05; ****, *P* < 0.0001. Abbreviations: CTG, CellTiter-Glo; LSD, least significant difference; Max, maximum; PO, per-oral; QD, once daily; QW, once weekly; RBC, red blood cell.

Previous studies have shown that DDR defects caused by LoF alterations in *ATM, BRCA1/2*, or several other genes associated with DNA repair lead to camonsertib sensitivity (6, 7). To evaluate whether gemcitabine further exacerbates the cytotoxicity of camonsertib in preclinical cell line models, an isogenic pair of *ATM*-proficient (WT) and deficient (*ATM-KO*) human RPE1-hTERT *TP53-KO* retinal epithelial cells was tested. Consistent with prior studies combining gemcitabine with an ATRi (10–13), gemcitabine exacerbated the cytotoxic effect of camonsertib at low nanomolar concentrations. In *ATM-KO* cells, the concentration of camonsertib required to suppress cell viability was markedly lower than in WT cells (Fig. 1A and B), confirming that *ATM* deficiency sensitizes cells to gemcitabine and camonsertib *in vitro*. We extended these findings to the *BRCA1*-deficient SUM149PT triple-negative breast cancer cell line (Fig. 1C), in which low doses of camonsertib and gemcitabine alone had only modest effects on cell viability but in combination led to near-complete inhibition of cell viability (Fig. 1C).

Subsequently, we explored the *in vivo* activity of the combination using low doses of camonsertib and gemcitabine in the *ATM-*deficient Granta-519 lymphoma and SUM149PT *BRCA1-*mutated breast cancer models. Immunodeficient mice bearing xenografts were treated with a vehicle control, camonsertib at 10 mg/kg (representing 1/3 of the mouse MTD), gemcitabine at 5, 10, or 20 mg/kg (less than 5%–20% of reportedly standard intraperitoneal dose and half as frequent; ref. 22), or their combination. The combination treatment led to a statistically significant and to a greater than 20% improved TGI response over single-agent activity (Fig. 1D; Supplementary Fig. S1A). The camonsertib and gemcitabine combination was well tolerated in mice, with no body weight loss observed (Supplementary Fig. S1B and S1C). On day 14, complete blood counts revealed no changes in red blood cells, a slight decrease in neutrophils, and an increase in platelets in the combination groups compared with both single-agent therapies (Fig. 1E). Taken together, preclinical studies suggested that lower doses of camonsertib and gemcitabine may be combined for antitumor activity while maintaining tolerability, providing a rationale for evaluating this combination clinically.

### TRESR module 4 clinical trial design, patient characteristics, and primary endpoints

The combination of camonsertib and gemcitabine was evaluated in module 4 of the TRESR study (NCT04497116). Seventy-six patients were enrolled across two arms (arm 1: *n* = 49 and arm 2: *n* = 27; Supplementary Fig. S2). Patients were initially enrolled into arm 1 to receive a combination of high-dose (1,000 mg/m^2^) gemcitabine with camonsertib at half the monotherapy RP2D (80 mg once daily). Arm 2 combined low doses of gemcitabine (<400 mg/m^2^) with camonsertib at 80 mg once daily or 120 mg once daily.

Baseline characteristics of the enrolled patients are shown in Table 1. The most common tumor types were ovarian (*n* = 41), pancreatic (*n* = 8), and breast (*n* = 6). The most frequent genetic alterations of those enrolled were *BRCA1* (*n* = 30), *BRCA2* (*n* = 20), and *ATM* (*n* = 14). Eighty percent of patients were female, the median age was 62 years (IQR: 55–69), and median lines of prior systemic therapies was 3 (IQR: 2–4.5), with preexposure rates to prior platinum of 89.5%, PARP inhibitor (PARPi) of 61.8%, and gemcitabine 25%.

**Table 1.** Summary of patient demographics and disease characteristics.

Characteristic	All patients (*N* = 76)
Sex, *n* (%)	​
Male	15 (19.7)
Female	61 (80.3)
Age (y)**,** median (range)	62 (39–94)
≥65 years	28 (36.8)
Race, *n*	​
White	57 (75)
Black or African American	5 (6.6)
Asian	5 (6.6)
Other	9 (11.8)
ECOG PS	​
0	31 (40.8)
1	45 (59.2)
Lines of prior systemic therapy	​
≤3	24 (31.6)
4 or more	52 (68.4)
Prior platinum	68 (89.5)
Prior PARPi	47 (61.8)
Prior PD-(L)1/inhibitor	18 (23.7)
Prior gemcitabine	19 (25)
Tumor type	​
Ovarian	41 (53.9)
Pancreatic	8 (10.5)
Breast	6 (7.9)
Other^a^	21 (27.6)
Enrollment gene	​
*ATM*	14 (18.4)
*BRCA1*	30 (39.5)
*BRCA2*	20 (26.3)
*CDK12*	2 (2.6)
*PALB2*	3 (3.9)
*SETD2*	2 (2.6)
Other^b^	5 (6.5)
Origin, *n* (%)	​
Germline	47 (61.8)
Somatic	25 (32.9)
Missing	4 (5.3)
Allelic status, *n* (%)	​
Biallelic	38 (50)
Nonbiallelic	13 (17.1)
Monoallelic	11 (14.5)
No loss	2 (2.6)
Unknown	25 (32.9)
Indeterminate	12 (15.8)
Missing	13 (17.1)

Note: Data cutoff of December 11, 2024.

Abbreviations: CARC, clear cell carcinoma; ECOG PS, Eastern Cooperative Oncology Group performance status; y, years.

a Other tumor types included ampullary (*n* = 1), cervical (*n* = 1), colorectal (*n* = 4), endometrial (*n* = 2), gastrointestinal (*n* = 1), head and neck (*n* = 1), kidney (*n* = 1), liver (*n* = 2), non–small cell lung (*n* = 3), mesothelioma (*n* = 1), prostate (*n* = 3), and uterine clear cell (*n* = 1).

b Other enrollment genes included *MRE11A* (*n* = 1), *RAD50* (*n* = 2), *RAD51B* (*n* = 1), and *RAD51C* (*n* = 1).

Dose finding started with enrollment in arm 1 at 80 mg once daily camonsertib [3/4d off; 2w on/1w off (2/1w)] with 1,000 mg/m^2^ gemcitabine (days 1 and 8) on a 21-day schedule. Due to poor tolerability, lower doses of gemcitabine (800, 600, and 400 mg/m^2^) were sequentially evaluated in subsequent cohorts, with no change to camonsertib starting dose or schedule. To manage cytopenia, gemcitabine was sequentially reduced to doses as low as 200 mg/m^2^. Based on the observed combinatorial activity of low-dose gemcitabine observed preclinically and to manage tolerability, arm 2 was opened to evaluate the combination of low-dose gemcitabine (100–200 mg/m^2^) with camonsertib on a 2 days on/5 days off schedule on either a 21-day (2/1w) or 28-day (1/1w) cycle (Supplementary Fig. S2). The 1/1w schedule was also evaluated in arm 1 as a dose optimization strategy.

Across all dose levels and schedules, neutropenia was the most common TRAE, with 64.5% (49/76) all grade and 50% (38/76) grade 3+ (Table 2). Other common TRAEs were anemia (59.2% all grades; 31.6% grade 3+), fatigue (57.9% all grades, 6.6% grade 3+), nausea (48.7% all grades, 0% grade 3+), alopecia (47.4% all grades, 0% grade 3+), and thrombocytopenia (35.5% all grades, 13.2% grade 3+). DLTs occurred in 36.5% (23/63) of evaluable patients, with neutropenia being the most common DLT [17.5% (11/63); Supplementary Table S3]. Typically, neutropenia occurred in the absence of fever, and recovery was rapid and did not require the administration of growth factors. Febrile neutropenia occurred in fewer than 5% of all patients (Table 2).

**Table 2.** TRAEs by schedule and grade in >5% of the total study population.

Preferred term	21-day schedule (*n* = 38)		28-day schedule (*n* = 38)		Total (*N* = 76)	
	All grades	Grade 3+	All grades	Grade 3+	All grades	Grade 3+
Patients with any TRAE, *n* (%)	38 (100)	33 (86.8)	38 (100)	26 (68.4)	76 (100)	59 (77.6)
Neutropenia	27 (71.1)	23 (60.5)	22 (57.9)	15 (39.5)	49 (64.5)	38 (50)
Anemia	23 (60.5)	15 (39.5)	22 (57.9)	9 (23.7)	45 (59.2)	24 (31.6)
Fatigue	23 (60.5)	2 (5.3)	21 (55.3)	3 (7.9)	44 (57.9)	5 (6.6)
Nausea	18 (47.4)	0	19 (50)	0	37 (48.7)	0
Alopecia	17 (44.7)	0	19 (50)	0	36 (47.4)	0
Platelet count decreased/ thrombocytopenia	18 (47.4)	8 (21.1)	9 (23.7)	2 (5.3)	27 (35.5)	10 (13.2)
Pyrexia	10 (26.3)	0	14 (36.8)	1 (2.6)	24 (31.6)	1 (1.3)
Vomiting	12 (31.6)	0	11 (28.9)	0	23 (30.3)	0
WBC count decrease/leukopenia	13 (34.2)	7 (18.4)	10(26.3)	4 (10.5)	23 (30.3)	11 (14.5)
Stomatitis	7 (18.4)	2 (5.3)	14 (36.8)	2 (5.3)	21 (27.6)	4 (5.3)
Diarrhea	7 (18.4)	1 (2.6)	10 (26.3)	0	17 (22.4)	1 (1.3)
Chills	8 (21.1)	0	6 (15.8)	0	14 (18.4)	0
Influenza-like illness	5 (13.2)	0	9 (23.7)	0	14 (18.4)	0
Decreased appetite	5 (13.2)	0	8 (21.1)	0	13 (17.1)	0
Headache	8 (21.1)	0	5 (13.2)	0	13 (17.1)	0
Pruritus	7 (18.4)	0	6 (15.8)	0	13 (17.1)	0
Alanine aminotransferase increased	7 (18.4)	1 (2.6)	5 (13.2)	2 (5.3)	12 (15.8)	3 (3.9)
Dysgeusia	5 (13.2)	0	5 (13.2)	0	10 (13.2)	0
Rash maculopapular	4 (10.5)	1 (2.6)	5 (13.2)	0	9 (11.8)	1 (1.3)
Aspartate aminotransferase increased	5 (13.2)	2 (5.3)	4 (10.5)	2 (5.3)	9 (11.8)	4 (5.3)
Dizziness	5 (13.2)	0	2 (5.3)	0	7 (9.2)	0
Dyspnea	5 (13.2)	0	2 (5.3)	0	7 (9.2)	0
Blood alkaline phosphatase increased	5 (13.2)	0	2 (5.3)	0	7 (9.2)	0
Constipation	2 (5.3)	0	4 (10.5)	0	6 (7.9)	0
Myalgia	3 (7.9)	0	3 (7.9)	0	6 (7.9)	0
Pain in extremity	4 (10.5)	1 (2.6)	2 (5.3)	0	6 (7.9)	1 (1.3)
Weight decreased	4 (10.5)	0	2 (5.3)	0	6 (7.9)	0
Abdominal pain	2 (5.3)	0	3 (7.9)	0	5 (6.6)	0
Abdominal pain upper	2 (5.3)	0	3 (7.9)	0	5 (6.6)	0
Arthralgia	3 (7.9)	0	2 (5.3)	0	5 (6.6)	0
Dry mouth	3 (7.9)	0	2 (5.3)	0	5 (6.6)	0
Lymphopenia	4 (10.5)	1 (2.6)	1 (2.6)	1 (2.6)	5 (6.6)	2 (2.6)
Muscular weakness	4 (10.5)	0	1 (2.6)	0	5 (6.6)	0
Palmar–plantar erythrodysesthesia syndrome	3 (7.9)	0	2 (5.3)	0	5 (6.6)	0
Back pain	3 (7.9)	0	1 (2.6)	0	4 (5.3)	0
Dehydration	3 (7.9)	0	1 (2.6)	0	4 (5.3)	0
Folliculitis	2 (5.3)	0	2 (5.3)	0	4 (5.3)	0
Gastroesophageal reflux disease	3 (7.9)	0	1 (2.6)	0	4 (5.3)	0
Mucosal inflammation	3 (7.9)	1 (2.6)	1 (2.6)	0	4 (5.3)	1 (1.3)
Rash	1 (2.6)	0	3 (7.9)	0	4 (5.3)	0

Note: Data cutoff date of December 11, 2024.

Abbreviation: WBC, while blood cell.

Treatment interruptions due to neutropenia occurred frequently [27.8% (5/18)] for patients in arm 1 treated on a 21-day cycle. To manage cytopenia, patients starting at higher gemcitabine doses often had sequential gemcitabine dose reductions to doses as low as 200 mg/m^2^. Introduction of the 1/1w schedule allowed for neutrophil recovery during scheduled weeks off, resulting in only 7.1% (2/28) of patients on a 28-day cycle experiencing a dose reduction due to neutropenia at the proposed expansion dose of 400 mg/m^2^ gemcitabine and 80 mg once daily (3/4d) camonsertib, whereas 47.4% (18/38) of patients on a 21-day cycle (all doses) required a dose reduction due to neutropenia (Supplementary Table S4). The DLT rate at the proposed expansion dose was 26.9% (7/26; Supplementary Table S3).

The PK profile of camonsertib at 80 and 120 mg revealed a dose-dependent trend in key parameters (Supplementary Table S5; Supplementary Fig. S3), consistent with previously observed PK characteristics of camonsertib (3).

### Secondary endpoints

**Figure 2.**
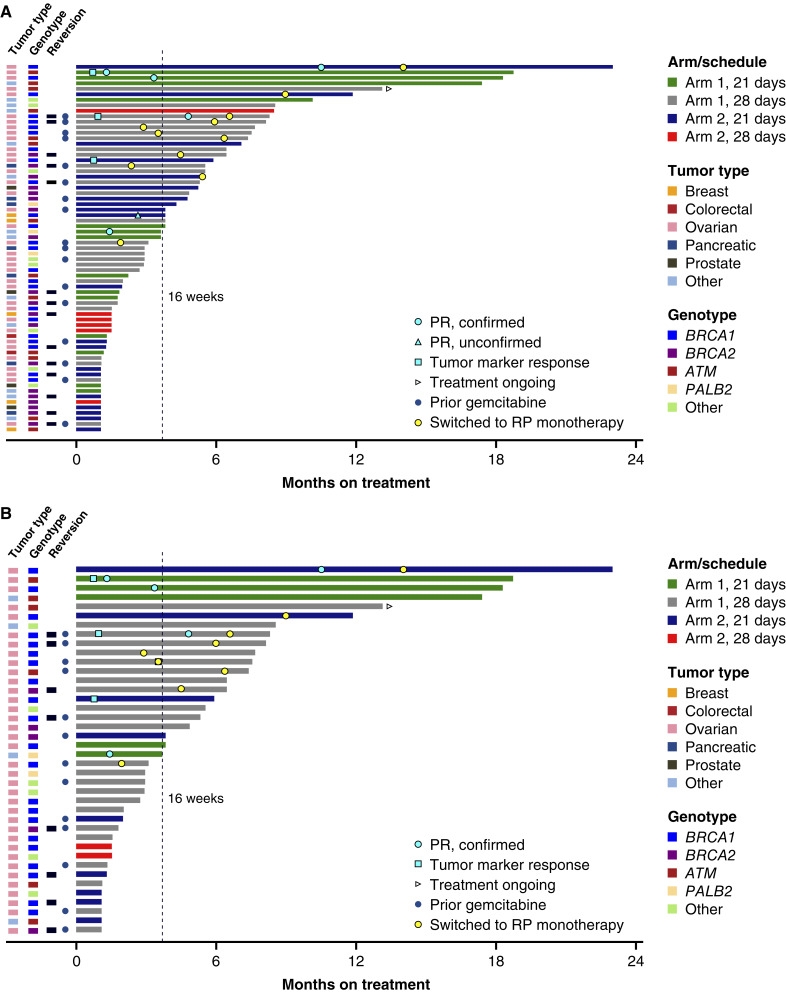
DOT for all patients and within the gynecologic subset by arm/schedule. Time on treatment by arm/schedule and tumor type for patients **A,** in all module 4 patients and **B,** within patients with gynecologic cancers.

Of the 76 patients enrolled, 67 were efficacy evaluable. The ORR (RECIST or CA-125) was 11.9% (8/67; Supplementary Table S6). RECIST v1.1 partial responses (PR) were observed in 10.6% (7/66; Fig. 2A; Supplementary Table S6) of patients: 6 confirmed (*n* = 5 ovarian, *n* = 1 endometrial) and 1 unconfirmed (breast); 1 additional patient with ovarian cancer had a confirmed CA-125 response by GCIG criteria, with no decline in target lesions (Table 3). Responses occurred and were maintained in patients treated at a range of gemcitabine starting doses (100–1000 mg/m^2^). Biomarker subgroups with responses included *ATM* (*n* = 1), *BRCA1* (*n* = 6), and *PALB2* (*n* = 1; Table 3). Across all tumors and genotypes, 44.8% (30/67; Supplementary Table S6) of patients derived clinical benefit (RECIST v1.1, TMR, or ≥16 weeks on treatment without progression); the median PFS (mPFS) was 19w (Supplementary Table S6). By treatment arm, the mPFS was 22.4w (arm 1) and 18w (arm 2; Supplementary Table S6). At the time of data cutoff, 1 patient remained on treatment; the duration of treatment (DOT) for all patients ranged from 1 to 98 + w.

**Table 3.** Confirmed responses in TRESR, module 4 patients.

Tumor type	Enrollment gene	Allelic status^a^	Number of prior lines, *n*; prior gemcitabine/PARPi/platinum, (Y/N)^b^	HRD status^c^	Starting gemcitabine dose, mg/m^2^	Final gemcitabine dose, mg/m^2^	DOT (w)	Best response	Best percent change in TL from baseline	MR (ctDNA)
Ovarian	*ATM*	Monoallelic	3; N/Y/Y-R	HRD	1,000	200	80	cPR	−52%	−74%
	*BRCA1*	Monoallelic	1; N/Y/Y	HRP	800	200	78	cPR	−31%	Not evaluable^d^
	*BRCA1*	Rearrangement	1; N/Y/Y	HRD	200	100	98	cPR	−32%	−88%
	*BRCA1*	Biallelic	8; Y/N/Y-UNK	HRD	400	200	32	cPR^e^	−43%	−90%
	*gBRCA1*	Indeterminate	6; Y/Y/Y-R	UNK	400	300	35	cPR^e^	−60%	Not evaluable^f^
	*gBRCA1*	Biallelic	3; N/Y/Y	HRD	100	100	25	CA-125	18%	Not tested
Endometrial	*gPALB2*	Indeterminate	4; N/N/Y	UNK	400	100	15	cPR	−64%	Not tested
Breast	*gBRCA1*	Indeterminate	5; N/Y/N	UNK	200	100	16	uPR^g^	−32%	Not tested

Note: Data cutoff date of December 11, 2024.

Abbreviations: cPR, confirmed PR; N, no; plat, platinum; QD, once daily; R, resistant; TL, tumor lesion; UNK, unknown; uPR, unconfirmed PR; Y, yes; Y-R, yes prior platinum–platinum-resistant.

a Reversions not detected in any patients with response.

b Prior platinum exposure is indicated with a Y, if patient is found to be platinum-resistant, it is indicated with an R.

c HRD status by SNIPDx HRD score.

d Not evaluable; did not pass minimum quality control parameters for MR analysis.

e Enrolled at proposed expansion dose.

f The first evaluable sample occurred outside the predetermined 12-week timeframe and was classified as not evaluable for MR.

g PR unconfirmed due to progression of brain lesions though sustained reduction in TLs.

Efficacy-evaluable patients with gynecologic cancers across all dose levels had an ORR of 17.5% (7/40), CBR of 50.0% (20/40) and mPFS of 32.6w (Fig. 2B; Supplementary Table S6). These patients were heavily pretreated (median 3.5 prior lines; IQR 3–5); the majority (72.5%; 29/40) had prior PARPi, and all patients received prior platinum treatment. At the proposed expansion dose, the ORR of patients with gynecologic cancers was 8.3% (2/24; Supplementary Table S6).

### Exploratory endpoints

Archival tissue was available from 74% (56/76) of enrolled patients, including from 71% (31/44) of patients enrolled based on tumor tissue analysis and from 81% (21/25) of patients enrolled based on a germline alteration. Local test results of DDR LoF alterations used for enrollment were retrospectively confirmed in archival tissue in 95% (53/56) of cases. The enrollment alteration was not confirmed in three patients with ovarian cancer, all of which were enrolled based on somatic *BRCA*1/2 homozygous deletions in tumor tissue that were determined to be single-copy losses. In retrospective liquid biopsy analysis (Guardant Infinity platform), the enrolling genetic alteration was confirmed in 82% of tested pretreatment samples (44/54). Reasons for nonconfirmation in liquid were as follows: one sample that failed quality control, six in which the alteration was not able to be detected in liquid biopsy [5 homozygous deletions or complex duplications (2 with confirmed single-copy loss in tissue) and 1 deep intronic variant], and last three samples with no result due to low ctDNA.

**Figure 3.**
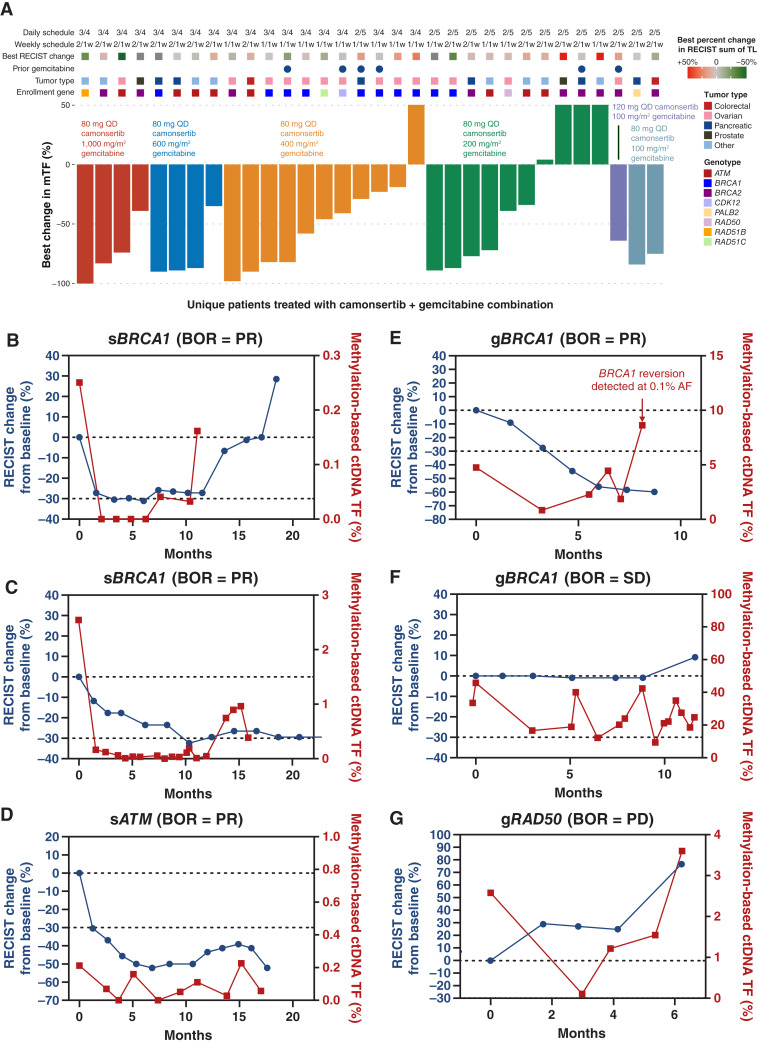
**A,** ctDNA MR by combination dose and schedule. Responses to low dose of gemcitabine in patients with ovarian cancer: **B,** BOR = PR, 80 mg camonsertib (2/5d) and 800 mg/m^2^ gemcitabine; **C,** BOR = PR, 80 mg camonsertib (3/4d) and 200 mg/m^2^ gemcitabine; **D,** BOR = PR, 80 mg camonsertib (3/4d) and 1,000 mg/m^2^ gemcitabine; **E,** BOR = PR, 80 mg camonsertib (3/4d) and 400 mg/m^2^ gemcitabine, 28 days; **F,** BOR = SD, 80 mg camonsertib (2/5d) and 200 mg/m^2^ gemcitabine, 21 days; **G,** BOR = PD, 80 mg camonsertib (3/4d) and 400 mg/m^2^ gemcitabine; 28 days. Abbreviations: 2/5d, 2 days on/5 days off; BOR, best overall response; TL, target lesion.

Among 35 patients with at least one pretreatment and one on-treatment plasma sample collected between weeks 4 and 12 of treatment, 91% (32/35) had a monitorable cTF at baseline (>0.1% cTF). An MR, defined as a ≥50% decline in cTF during treatment compared with baseline, was observed in 61% (22/36) of patients with a median time to best MR of 6.1w (range 4–12 weeks). MR rates were comparable between the cohorts treated with 400 mg/m^2^ and 200 mg/m^2^ gemcitabine when combined with 80 mg camonsertib (5/11 and 4/10, respectively; Fig. 3A). All three patients treated with low doses of 100 mg/m^2^ gemcitabine in combination with camonsertib also displayed MRs.

cTF was followed longitudinally to progression for six patients with ovarian cancer having a DOT ≥6 months: four with RECISTv1.1 PRs, one with 1 year of stable disease (SD), and one with early progressive disease (PD) but remained on treatment for 6 months (Fig. 3B). In the four patients with PRs, early ctDNA declines were observed and trends in cTF qualitatively followed that of RECIST v1.1 sum of target lesions, all in the setting of multiple dose reductions in gemcitabine (Fig. 3B–E). In the patient with approximately 1 year of SD, the patient started with a high cTF (∼50%) which declined; however, cTF remained quantifiable through treatment (Fig. 3F). In the patient with a best response of PD, target lesions initially increased and then stabilized for approximately 3 months; during which time there was a sharp decline in cTF. A subsequent increase in cTF 1 month later preceded the rebound in target lesions. (Fig. 3G).

As the majority of patients enrolled had a *BRCA*-associated tumor type (ovarian, pancreatic, breast, and prostate) with DDR gene alterations, nearly all patients had prior treatment with a PARPi or platinum. We therefore investigated the presence of baseline reversions within the four core *BRCA1/2*-associated tumor types harboring enrollment alterations on HR-associated genes: *BRCA1/2*, *PALB2*, or *RAD51* paralogs. Reversions are secondary genetic alterations that restore the open reading frame of the enrollment gene, thereby eliminating the synthetic lethal interaction, and are established as predictive biomarkers of PARPi and platinum resistance (23–26). Within this subset of patients (*N* = 45), reversions were detected in 13 (29%) patients (7 in plasma, 5 in archival tissue, and 1 in both). Across all dose levels, patients with reverted tumors had similar median DOT of 1.5 months when compared with 2.9 months without reversions (log-rank, *P* = 0.24; Supplementary Fig. S4A) and a mPFS of 4 versus 4.2 months (log-rank, *P* = 0.099; Supplementary Fig. S4B). In a patient treated with prior platinum and PARPi harboring a g*BRCA1*-mutated ovarian tumor, a reversion was not present at baseline yet appeared at the time of progression after receiving 30 weeks of gemcitabine and camonsertib treatment (Fig. 3E; Supplementary Fig. S4C). Whether the reversion was present but undetectable prior to starting treatment or arose in response to the combination therapy cannot be determined.

Outside of this subset, we identified one additional reversion in a pretreatment liquid biopsy from a patient with HER2-positive breast cancer enrolled based on a premature stop codon in the *ATM* gene (p.R250*) detected in an archival sample. Retrospective analysis confirmed that the enrolling alteration was biallelic, with corresponding ATM IHC loss in a breast biopsy obtained before initiating carboplatin. This patient’s best response was SD, with a 12% increase in target lesions, and therapy was discontinued after 4 weeks.

## Discussion

The rationale for the combination of gemcitabine with an ATRi was based on complementary mechanisms of promoting replication stress and impairment of the DDR, ultimately resulting in enhanced DNA damage incompatible with cell viability (27). Previous work has established the benefit of combining an ATRi with gemcitabine in cell lines, tumor xenograft models, and clinical trials (10–16). We demonstrated that gemcitabine potentiates camo*nsertib cytotoxicity in vitro and enhances preclinical efficacy with the combination* in vivo in the setting of DDR deficiency, thereby providing rationale for the phase I study to evaluate this combination in DDR-deficient tumors, including the utilization of low doses of gemcitabine to manage toxicities.

Clinically, the greatest benefit was seen in patients with gynecologic malignancies, particularly those harboring *BRCA1* alterations or signatures of HR deficiency (HRD), although one partial responder enrolled with a monoallelic *BRCA1* alteration was retrospectively found to be HR-proficient (HRP; ref. 15). A review of nine phase I trial outcomes in biomarker-driven selection of ovarian cancer therapies showed a large range in ORR (9.6%–47.1%) which varied significantly depending on the drug type, the patient's molecular profile, and prior treatments received (28). Furthermore, another analysis of patients with ovarian cancer in phase I trials found that for those patients who progressed on PARP, response rates were low, 14.7%, particularly if heavily pretreated (29). Notably, multiple patients enrolled across both study arms remained on treatment for >12 months with sustained disease control despite treatment with individually subtherapeutic levels of gemcitabine and camonsertib. A proposed expansion dose was evaluated at 80 mg once daily camonsertib (3/4d schedule) with 400 mg/m^2^ gemcitabine (days 1 and 15) on a 28-day, 1/1w cycle. The primary observed toxicity at the proposed expansion dose was neutropenia, though the biweekly dosing allowed for recovery in the off-weeks and resulted in fewer dose modifications. Although the intermittent weekly dosing schedule improved tolerability, clinical efficacy was not optimal with this less intensive dosing regimen, which indicates that further dose and schedule optimization is warranted to establish the RP2D. Due to persistent low-grade tolerability issues associated with gemcitabine which occurred even at low doses, a subset of patients discontinued gemcitabine and continued camonsertib monotherapy at the RP2D (160 mg once daily 3/4d, 2/1w; ref. 30).

High concordance between local and central genomic testing was observed; however, genomic analysis of responders (Table 3) highlights the complexity of diagnosing DDR LoF alterations and the difficulty in identifying optimal predictive biomarkers for this drug combination and for other DDR-targeting agents. In one case, a patient with ovarian cancer was enrolled based on a monoallelic LoF alteration and was initially suspected to be HRP; however, the patient was retrospectively determined to have HRD scarring. Another patient enrolled with a *BRCA1* LOF alteration, that was unexpectedly found to be monoallelic, and consistent with this, no signature of HRD was detected. Detecting complex alterations in both liquid biopsy and tissue remains a challenge, particularly in the accurate identification of rearrangements and homozygous deletions (31, 32). These findings underscore the limitations of current clinically utilized genomic tests and highlight the need for continued improvement in genomic profiling, especially for the detection of LoF alterations, and the need to develop improved functional assays of HR.

As with any drug combination, one key challenge is to define a strategy that efficiently utilizes small cohorts to refine the necessary dose and schedule to balance tumor control with drug tolerability. As previously shown, in the phase I dose-finding study for camonsertib monotherapy in TRESR, we evaluated the totality of safety, tolerability, and efficacy signals, including ctDNA MR, which ultimately supported the RP2D that balanced the risk profile, in line with Project Optimus (30). The technical challenge with ctDNA evaluation is that many patients do not harbor detectable somatic variants in blood or do not have available tissue for a tumor-informed approach. Therefore, we estimated ctDNA TF by assessing differentially methylated regions in cell-free DNA which demonstrated high sensitivity, as the majority (>90%) of patients were monitorable. In contrast, in a prior analysis of patients enrolled in the camonsertib monotherapy module of the TRESR study, which had identical eligibility criteria, only 64% of patients were monitorable using a commercially available comprehensive genomic profiling liquid biopsy panel (3). In this study, a trend of greater declines in ctDNA were observed in the 400 mg/m^2^ versus 200 mg/m^2^ gemcitabine doses in combination with camonsertib, which is consistent with the observed increase in mPFS in arm 1 compared with arm 2. These results provide additional data in support of the proposed expansion dose.

Many patients treated in this study had received prior platinum and PARPi treatment, leading us to hypothesize that several patients may harbor secondary alterations, or “reversions,” that restore the functional gene sequence of the primary DDR LoF alteration eliminating the synthetic lethal interaction and allow better contextualization of outcomes. Although reversions are a validated, negative predictive biomarker of response to PARPi and platinum, their relevance in the context of ATR inhibition is less clear and for these reasons, and the practical challenges in contemporaneous detection, were not excluded from trial entry. In our prior analysis of patients with reversions (*n* = 10) treated with camonsertib monotherapy, there was one RECIST response and several patients with protocol-defined clinical benefit (≥16 weeks on treatment; ref. 3). The small sample size of this study limits our ability to draw definitive conclusions about outcomes in these patients with reversions, which is confounded by the technologic challenge of detecting these alterations (23, 26). Many reversions are likely to remain undetected due to the coverage limitations of NGS panels and the inherent challenges in identifying deletions and rearrangements. As previously described, one reversion was detected in the *ATM* gene in this cohort (20). Here, we present the clinical outcome, and despite the lack of benefit observed, additional clinical cases will need to be identified to clarify the potential impact of reversions in this gene. Understanding the predictive and prognostic significance of reversion alterations as well as other relevant co-occurring alterations (e.g., those associated with replication stress) will require larger further investigation.

This study has several limitations, beyond those applicable to all phase I dose-escalation studies. Preclinically, we tested the camonsertib with gemcitabine combination in a *BRCA1*-deficient breast cancer cell model and an ATM-mutated mantle cell lymphoma xenograft, as well as an isogenic *ATM* WT and KO nonmalignant immortalized cell system. Studying this combination in DDR-deficient models of gynecologic cancers would be of high interest with regards to the relevant patient population in our clinical study. In the clinical trial, responses occurred and were maintained after gemcitabine dose reductions, but the potential influence of the starting dose remains uncertain, as the high starting dose may introduce a priming effect. Furthermore, initial enrollment in arm 1 excluded patients with prior gemcitabine exposure, raising the possibility that the observed benefit at high starting doses could be, at least in part, attributed to gemcitabine alone.

In summary, the phase I results included here evaluating the safety, tolerability, and efficacy of low-dose camonsertib and gemcitabine provide further data on schedule and dosing for this DDR combination. However, achieving an optimal therapeutic balance remains a challenge due to hematologic toxicity even at low doses of gemcitabine. A subset of heavily pretreated patients with few or no available treatment options clearly gained clinical benefit on this trial, which may justify the use of this drug combination with this side effect profile in this context. Future investigation is necessary to molecularly characterize this subset of patients to improve patient selection to ensure sensitivity of tumors to this drug combination. To manage this hematologic toxicity, future studies could incorporate prophylactic growth factor administration and should ensure close monitoring of hematologic toxicities. Ongoing efforts to refine patient selection criteria, optimize dosing strategies, and integrate biomarker-driven approaches will be crucial to maximizing the clinical potential of ATRis in combination regimens.

## Supplementary Material

Supplementary Figure S1 Mice bearing SUM149PT (A and C) or Granta-519 (B) tumors were treated with camonsertib (3/4d, weekly) or gemcitabine (QW) as single agents and in combination at the doses indicated. A) Tumor growth is represented as mean ± SEM; n = 7 mice/group; TGI = tumor growth inhibition (%). B, C) Body weights are represented as mean change relative to Day 0 ± SEM. Statistical differences between groups were determined using an unpaired t-test with Welch’s correction. *P < .05; **P < .01 (GraphPad Prism v10.4).

Supplementary Figure S2 Study schematic for TRESR, Module 4.

Supplementary Figure S3 Camonsertib concentration levels at 80 and 120 mg – cycle 1 day 1

Supplementary Figure S4 Kaplan-meier estimate for A, DOT and B, PFS in patients with and without reversions enrolled on LoF in BRCA1/2, RAD51p, or PALB2 in HRD associated tumor types (breast, prostate, pancreatic, ovarian) C) On-treatment BRCA1 reversion detected (RECIST and ctDNA cTF in Fig. 3D)

Supplementary Table S1 Institutional Review Boards of participating institutions

Supplementary Table S2 Study representativeness table

Supplementary Table S3 Treatment-emergent DLT adverse events

Supplementary Table S4 TRAEs leading to dose reductions and/or interruptions

Supplementary Table S5 Drug combination pharmacokinetic parameters

Supplementary Table S6 Additional efficacy parameters

## Data Availability

To minimize the risk of patient reidentification, data will only be shared upon reasonable request. For eligible studies, qualified researchers may request access to individual patient-level clinical data and additional raw biomarker data by emailing Repare Therapeutics, Inc (info@reparerx.com). Once approved, the data will be shared through a secure data sharing link.
