# Supplementary material for: Camonsertib, an ATRi, in Combination with Low-Dose Gemcitabine in Solid Tumors with DNA Damage Response Aberrations: Preclinical and Phase Ib Results
Source: Clin Cancer Res. 2026 Jan 21;32(8):1411–23. doi: 10.1158/1078-0432.CCR-25-2240 (PMC13080318; doi:10.1158/1078-0432.CCR-25-2240)
Supplement: Supplementary Figure S1 — Mice bearing SUM149PT (A and C) or Granta-519 (B) tumors were treated with camonsertib (3/4d, weekly) or gemcitabine (QW) as single agents and in combination at the doses indicated. A) Tumor growth is represented as mean ± SEM; n = 7 mice/group; TGI = tumor growth inhibition (%). B, C) Body weights are represented as mean change relative to Day 0 ± SEM. Statistical differences between groups were determined using an unpaired t-test with Welch’s correction. *P < .05; **P < .01 (GraphPad Prism v10.4). [file ccr-25-2240_supplementary_figure_s1_suppfs1.docx]

**
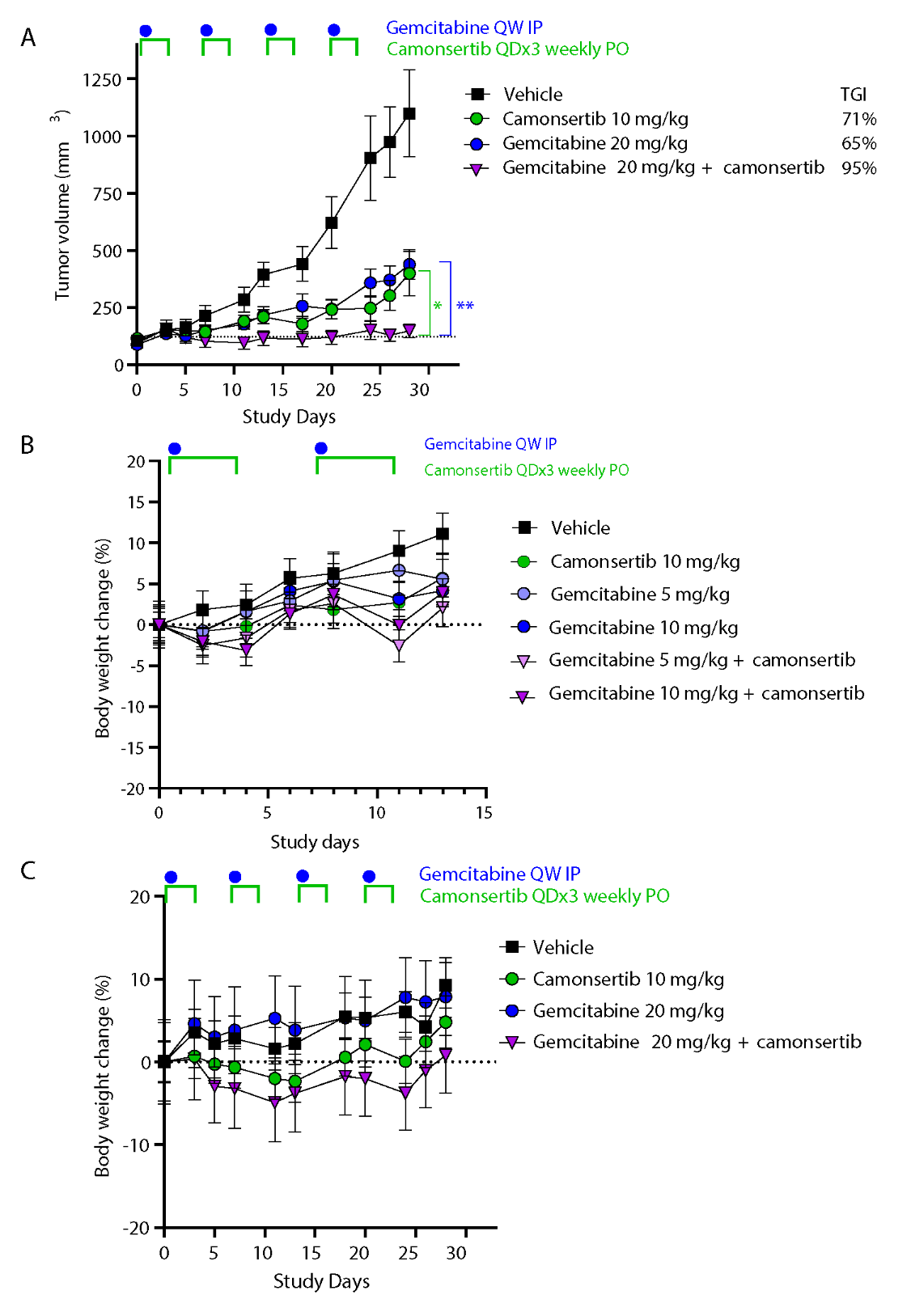
**

**Supplementary Fig. S1**. Mice bearing SUM149PT (**A** and **C**) or Granta-519 (**B**) tumors were treated with camonsertib (3/4d, weekly) or gemcitabine (QW) as single agents and in combination at the doses indicated. **A)** Tumor growth is represented as mean ± SEM; n=7 mice/group; TGI (%). **B, C)** Body weights are represented as mean change relative to Day 0 ± SEM. Statistical differences between groups were determined using an unpaired t-test with Welch’s correction. **P*<.05; ***P*<.01 (GraphPad Prism v10.4).

IP, intraperitoneal; PO, per oral; TGI, tumor growth inhibition; QD, once daily; QW, once weekly.
