# Supplementary material for: Camonsertib, an ATRi, in Combination with Low-Dose Gemcitabine in Solid Tumors with DNA Damage Response Aberrations: Preclinical and Phase Ib Results
Source: Clin Cancer Res. 2026 Jan 21;32(8):1411–23. doi: 10.1158/1078-0432.CCR-25-2240 (PMC13080318; doi:10.1158/1078-0432.CCR-25-2240)
Supplement: Supplementary Figure S2 — Study schematic for TRESR, Module 4. [file ccr-25-2240_supplementary_figure_s2_suppfs2.docx]

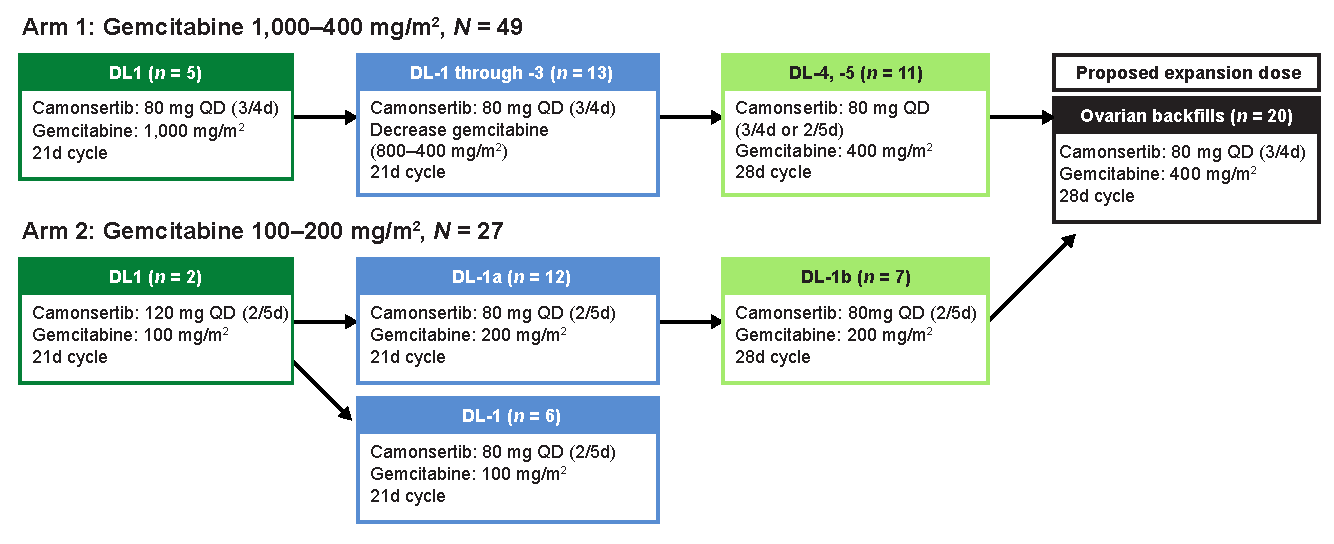


## **Supplementary Fig. S2.** Study schematic for TRESR, Module 4.

2/5d, 2 days on/5 days off; 3/4d, 3 days on/4 days off; d, day; DL, dose level; QD, once daily; RP2D, recommended Phase II dose
