# Supplementary figures and images for: Camonsertib, an ATRi, in Combination with Low-Dose Gemcitabine in Solid Tumors with DNA Damage Response Aberrations: Preclinical and Phase Ib Results
Source: Clin Cancer Res. 2026 Jan 21;32(8):1411–23. doi: 10.1158/1078-0432.CCR-25-2240 (PMC13080318; doi:10.1158/1078-0432.CCR-25-2240)

**
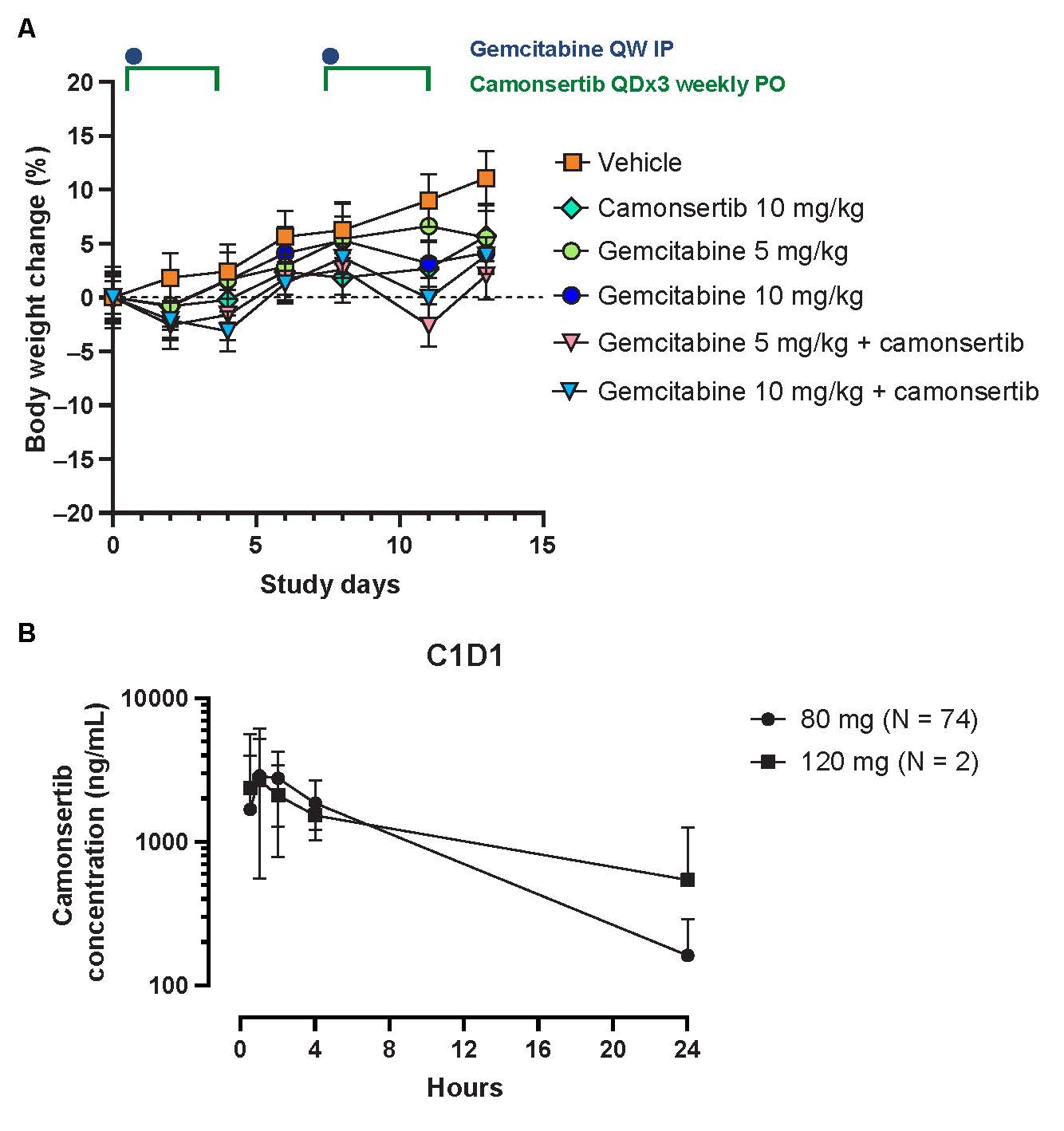
**

## **Supplementary Fig. S3.** Camonsertib concentration levels at 80 mg and 120 mg – cycle 1 day 1

Supplement: Supplementary Figure S3 — Camonsertib concentration levels at 80 and 120 mg – cycle 1 day 1 [file ccr-25-2240_supplementary_figure_s3_suppfs3.docx]
