# Supplementary material for: Camonsertib, an ATRi, in Combination with Low-Dose Gemcitabine in Solid Tumors with DNA Damage Response Aberrations: Preclinical and Phase Ib Results
Source: Clin Cancer Res. 2026 Jan 21;32(8):1411–23. doi: 10.1158/1078-0432.CCR-25-2240 (PMC13080318; doi:10.1158/1078-0432.CCR-25-2240)
Supplement: Supplementary Figure S4 — Kaplan-meier estimate for A, DOT and B, PFS in patients with and without reversions enrolled on LoF in BRCA1/2, RAD51p, or PALB2 in HRD associated tumor types (breast, prostate, pancreatic, ovarian) C) On-treatment BRCA1 reversion detected (RECIST and ctDNA cTF in Fig. 3D) [file ccr-25-2240_supplementary_figure_s4_suppfs4.docx]

**
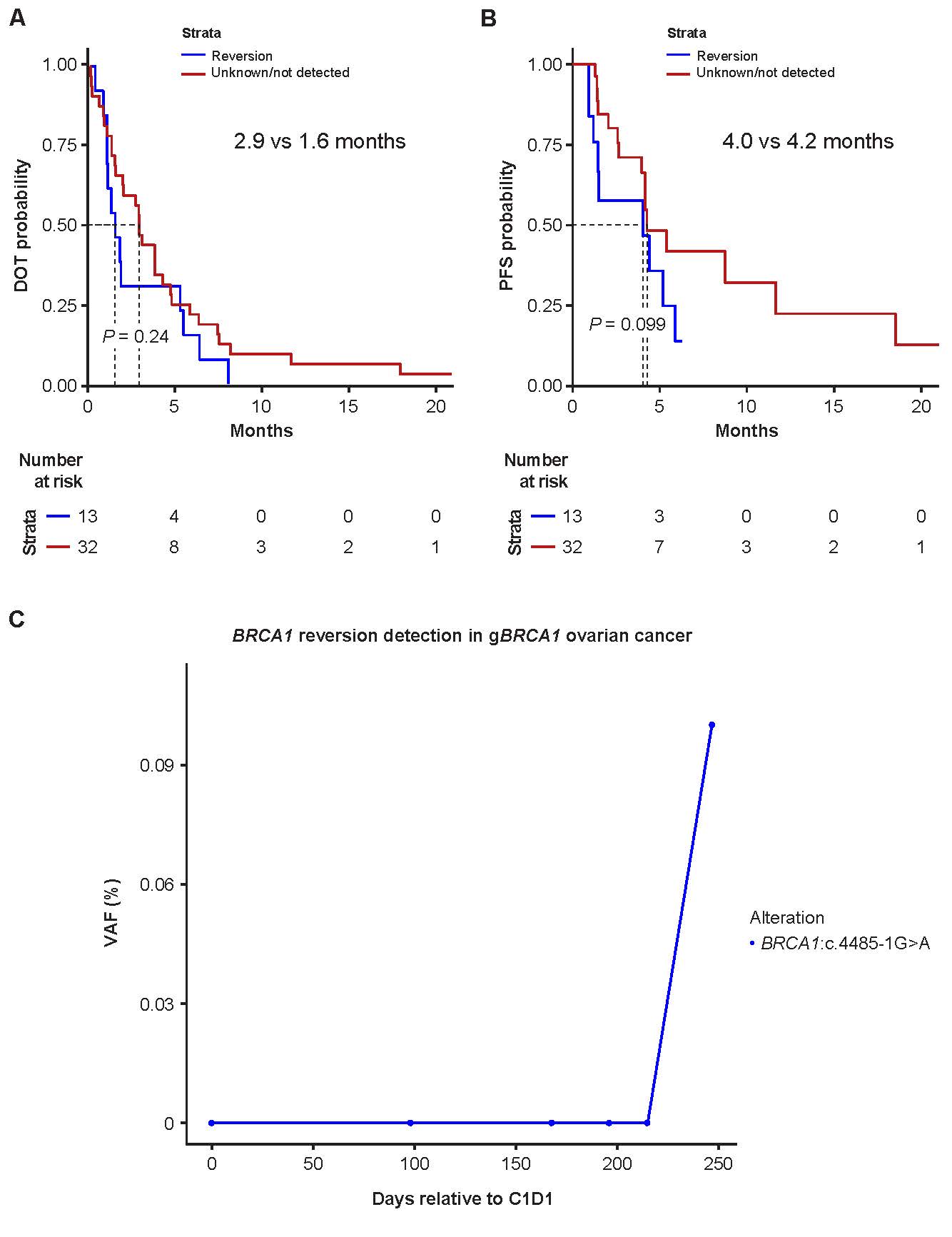
**

## **Supplementary Fig. S4:** Kaplan-meier estimate for **A,** DOT and **B,** PFS in patients with and without reversions enrolled on LoF in *BRCA1/2*, *RAD51p*, or *PALB2* in HRD associated tumor types (breast, prostate, pancreatic, ovarian) **C)** On-treatment *BRCA1* reversion detected (RECIST and ctDNA cTF in Fig. 3D)

C1D1, cycle 1 day 1; ctDNA, circulating tumor DNA; cTF, circulating tumor fraction; DOT, duration of treatment; HRD, Homologous recombination deficiency; PFS, progression-free survival; RECIST, Response Evaluation Criteria in Solid Tumors v1.1; VAF, variant allele frequency.
