# Supplementary material for: Camonsertib, an ATRi, in Combination with Low-Dose Gemcitabine in Solid Tumors with DNA Damage Response Aberrations: Preclinical and Phase Ib Results
Source: Clin Cancer Res. 2026 Jan 21;32(8):1411–23. doi: 10.1158/1078-0432.CCR-25-2240 (PMC13080318; doi:10.1158/1078-0432.CCR-25-2240)
Supplement: Supplementary Table S1 — Institutional Review Boards of participating institutions [file ccr-25-2240_supplementary_table_s1_suppts1.docx]

| Supplementary Table S1. Institutional Review Boards of participating institutions | | |
| --- | --- | --- |
| **TRESR (NCT04497116)** | | |
| **IRB name** | **Affiliation(s)** | **Country** |
| University of Texas MD Anderson Cancer Center Institutional Review Boards | MD Anderson Cancer Center | USA |
| Dana Farber Cancer Institute Institutional Review Boards | Dana-Farber Cancer Center | USA |
| Advarra IRB, Inc. | Sarah Cannon Research Institute – TN | USA |
| Memorial Sloan-Kettering Cancer Center Institutional Review Board/Privacy Board | Memorial Sloan Kettering Cancer Center | USA |
| Duke University Health Systems Institutional Review Boards | Duke Cancer Institute | USA |
| Rhode Island Hospital IRB | Rhode Island Hospital | USA |
| Northwestern University IRB Panels A, B, C, D and Q | Robert H. Lurie Comprehensive Cancer Center of Northwestern University | USA |
| University Health Network Research Ethics Board | Princess Margaret Cancer Centre | Canada |
| Health Research Authority, North East – Tyne and Wear South Research Ethics Committee | The Christie NHS Foundation Trust Manchester    Freeman Hospital Newcastle/Sir Bobby Robson Cancer Trials Research Centre    Sarah Cannon Research Institute – London | United Kingdom |
| Scientific Ethics Committees for the Capital Region (Denmark) – *translated name* | Rigshospitalet, University Hospital of Copenhagen | Denmark |
