# Supplementary material for: Camonsertib, an ATRi, in Combination with Low-Dose Gemcitabine in Solid Tumors with DNA Damage Response Aberrations: Preclinical and Phase Ib Results
Source: Clin Cancer Res. 2026 Jan 21;32(8):1411–23. doi: 10.1158/1078-0432.CCR-25-2240 (PMC13080318; doi:10.1158/1078-0432.CCR-25-2240)
Supplement: Supplementary Table S2 — Study representativeness table [file ccr-25-2240_supplementary_table_s2_suppts2.docx]

| **Supplementary Table S2.** Representativeness of study population | |
| --- | --- |
| Cancer types | Advanced or metastatic solid tumors with loss of function in select DNA damage response (DDR) genes |
| Considerations related to: | |
| Sex | Globally, an estimated 18.7 million cancer cases occurred in 2022, with 9.57 million (51.0%) cases occurring in men and 9.18 million (49.0%) cases occurring in women.^1^ |
| Age | The median age at diagnosis of any cancer in the U.S. is 67 years. Between 2018 and 2022, 30.5% of all cancers were diagnosed among people aged 65–74 years.^2^ |
| Race/Ethnicity | Between 2018 and 2022 in the U.S., the rate of new cancer cases per 100,000 was highest in White patients (477.9) and lowest in Asian/Pacific islander patients (311.3). During a similar time period of 2019 to 2023, cancer death rates per 100,000 were highest in black patients (166.5) and lowest in Asian/Pacific islander patients (93.1).^2^ |
| Geography | Cancer ranks as the second most common cause of death worldwide. |
| Other considerations | The most common tumor types in TRESR Module 4 were ovarian (54%), pancreatic (11%), and breast (8%). The most common enrollment genes were *ATM* (18%), *BRCA1* (40%), and *BRCA2* (26%). |
| Overall representativeness of this study | The population in this present study was a subset of the overall TRESR population, with generally comparable demographics and included patients in the US, Canada, Denmark, and United Kingdom. The proportion of female patients (80% in TRESR Module 4) were higher compared to the global average of 49.0%,^1^ due to backfill cohorts for patients with ovarian cancer which were opened after initial efficacy was observed in gynecological cancers resulting in higher representation of female patients. The median age of patients from TRESR Module 4 was 62 lower than the median age of 67 years for all cancers in the US ^2^ but is comparable to the recently reported median age of phase 1 clinical trial participants in the U.S. between 2000 and 2018 of 59 years.^3^  Within Module 4 of the TRESR study, Caucasians represented >50% of the study population with both African American and Asians representing 6.6% of the population each. Race was not reported in roughly 10% of the study population (data on file). For comparison, phase 1 clinical trial participants in the U.S. between 2000 and 2018 were 86.1% White, 4.5% Asian and 6.2% Black.^3^  As a Phase 1 trial with eligibility primary based on genomic alterations, TRESR included a heavily pre-treated population of patients with complex, heterogenous disease. The predominance of alterations in *ATM* and *BRCA1/2* in TRESR, and specifically in Module 4, is generally in line with reported relative frequencies of these alterations in pan-cancer and tumor-type-specific analyses. The prevalence of loss-of-function alterations in DDR genes other than *ATM* and *BRCA1/2* tend to be very low in advanced and metastatic cancers. |

1. World Cancer Research Fund International: Worldwide cancer data. Available at [Worldwide cancer data | World Cancer Research Fund International (wcrf.org)](https://www.wcrf.org/cancer-trends/worldwide-cancer-data/) [accessed 16 June 2025]
2. National Cancer Institute Surveillance, Epidemiology and End Results (SEER) program. Cancer Stat Facts: Cancer of Any Site. Available at [Cancer of Any Site — Cancer Stat Facts](https://seer.cancer.gov/statfacts/html/all.html) [accessed 16 June 2025]
3. Dunlop H, et al. JAMA Netw Open. 2022;5(11):e2239884. doi:10.1001/jamanetworkopen.2022.39884
