# Supplementary material for: Camonsertib, an ATRi, in Combination with Low-Dose Gemcitabine in Solid Tumors with DNA Damage Response Aberrations: Preclinical and Phase Ib Results
Source: Clin Cancer Res. 2026 Jan 21;32(8):1411–23. doi: 10.1158/1078-0432.CCR-25-2240 (PMC13080318; doi:10.1158/1078-0432.CCR-25-2240)
Supplement: Supplementary Table S3 — Treatment-emergent DLT adverse events [file ccr-25-2240_supplementary_table_s3_suppts3.docx]

| Supplementary Table S3. Treatment-emergent DLT adverse events | | | | | | | | | | | |
| --- | --- | --- | --- | --- | --- | --- | --- | --- | --- | --- | --- |
|  | **Arm 1**  **(N=39)** | | | | |  | **Arm 2**  **(N=24)** | | | | |
|  | **Camonsertib 80mgQD/ Gemcitabine 600mg, 3/4d, 2/1w**  **(*n* = 2)** | **Camonsertib 80 mg QD/ Gemcitabine 1000 mg, 3/4d, 2/1w  (*n* = 3)** | **Camonsertib 80 mg QD/ Gemcitabine 400 mg, 3/4d, 2/1w  (*n* = 5)** | **Proposed expansion dose**  **Camonsertib 80 mg QD/ Gemcitabine 400 mg, 3/4d, 1/1w  (*n* = 26)** | **Camonsertib 80 mg QD/ Gemcitabine 400 mg, 2/5d, 1/1w  (*n* = 3)** |  | **Camonsertib 120mg QD/ Gemcitabine 100mg, 2/5d, 2/1w (*n* = 2)** | **Camonsertib 80 mg QD/ Gemcitabine 100 mg, 2/5d, 2/1w  (*n* = 6)** | **Camonsertib 80 mg QD/ Gemcitabine 200 mg, 2/5d, 2/1w  (*n* = 9)** | **Camonsertib 80 mg QD/ Gemcitabine 200 mg, 2/5d, 1/1w  (*n* = 7)** | **Total  (*N* = 63)** |
| Any DLT event, *n* (%) | 1 (50.0) | 2 (66.7) | 5 (100.0) | 7 (26.9) | 2 (66.7) |  | 1 (50.0) | 2 (33.3) | 3 (33.3) | 0 | 23 (36.5) |
| Neutrophil count decreased/ Neutropenia | 1 (50.0) | 1 (33.3) | 2 (40.0) | 3 (11.5) | 1 (33.3) |  | 0 | 1 (16.7) | 2 (22.2) | 0 | 11 (17.5) |
| Anemia | 0 | 1 (33.3) | 1 (20.0) | 0 | 0 |  | 0 | 1 (16.7) | 0 | 0 | 3 (4.8) |
| Aspartate aminotransferase increased | 0 | 0 | 0 | 2 (7.7) | 0 |  | 0 | 0 | 1 (11.1) | 0 | 3 (4.8) |
| Febrile neutropenia | 0 | 1 (33.3) | 0 | 0 | 0 |  | 1 (50.0) | 0 | 0 | 0 | 2 (3.2) |
| Alanine aminotransferase increased | 0 | 0 | 0 | 1 (3.8) | 0 |  | 0 | 0 | 0 | 0 | 1 (1.6) |
| Fatigue | 0 | 0 | 0 | 1 (3.8) | 0 |  | 0 | 0 | 0 | 0 | 1 (1.6) |
| Mucosal inflammation | 0 | 0 | 1 (20.0) | 0 | 0 |  | 0 | 0 | 0 | 0 | 1 (1.6) |
| Pneumonitis | 0 | 0 | 0 | 0 | 1 (33.3) |  | 0 | 0 | 0 | 0 | 1 (1.6) |
| Rash maculo-papular | 0 | 0 | 1 (20.0) | 0 | 0 |  | 0 | 0 | 0 | 0 | 1 (1.6) |
| Stomatitis | 0 | 0 | 0 | 1 (3.8) | 0 |  | 0 | 0 | 0 | 0 | 1 (1.6) |

Note: Data cut-off date of December 11, 2024 for all treated patients who were DLT evaluable (N=63).

1/1w, 1 week on/1 week off; 2/1w, 2 weeks on/1 week off; 2/5d, 2 days on/5 days off; 3/4d, 3 days on/4 days off; AE, adverse event; DLT, dose-limiting toxicity; QD, once daily; TRAE, treatment-related adverse event; RP2D, recommended phase II dose; w, week
