# Supplementary material for: Camonsertib, an ATRi, in Combination with Low-Dose Gemcitabine in Solid Tumors with DNA Damage Response Aberrations: Preclinical and Phase Ib Results
Source: Clin Cancer Res. 2026 Jan 21;32(8):1411–23. doi: 10.1158/1078-0432.CCR-25-2240 (PMC13080318; doi:10.1158/1078-0432.CCR-25-2240)
Supplement: Supplementary Table S4 — TRAEs leading to dose reductions and/or interruptions [file ccr-25-2240_supplementary_table_s4_suppts4.docx]

| **Supplementary Table S4:** TRAEs leading to dose reductions/interruptions for any study drug | | | | | | | | | | | |
| --- | --- | --- | --- | --- | --- | --- | --- | --- | --- | --- | --- |
|  |  | Arm 1 | |  | Arm 2 | |  | Proposed expansion dose^a^ |  | All doses/schedules | |
|  |  | Gemcitabine  1000–400 mg/m^2^ | |  | Gemcitabine  100–200 mg/m^2^ | |  | Camonsertib 80mg QD/  Gemcitabine 400mg, 3/4d, 1/1w |  |  | |
|  |  | **21-Day**  (*N* = 18) | **28-Day**  (*N* = 31) |  | **21-Day**  (*N* = 20) | **28-Day**  (*N* = 7) |  | **28-Day**  (*N* = 28) |  | **21-Day**  (*N* = 38) | **28-Day**  (*N* = 38) |
| **Interruption^b^** | Any event | 13 (72.2) | 7 (22.6) |  | 12 (60.0) | 0 |  | 6 (21.4) |  | 25 (65.8) | 7 (18.4) |
|  | Neutropenia | 5 (27.8) | 0 |  | 6 (30.0) | 0 |  | 0 |  | 11 (28.9) | 0 |
| **Reduction^b^** | Any event | 14 (77.8) | 9 (29.0) |  | 12 (60.0) | 0 |  | 8 (28.6) |  | 26 (68.4) | 9 (23.7) |
|  | Neutropenia | 11 (61.1) | 2 (6.5) |  | 7 (35.0) | 0 |  | 2 (7.1) |  | 18 (47.4) | 2 (5.3) |

Note: Data cut-off date of December 11, 2024.

^a^ Included all patients from Arm 1, 28-day column excluding 3 patients treated with gemcitabine on the 2/5d schedule (Supplementary Fig. S2).

^b^ If a patient received both a dose interruption and a dose reduction, the AE event was tabulated as a dose reduction only.

1/1w, 1 weeks on/1 week off; 2/5, 2 days on/5 days off; 3/4, 3 days on/4 days off; AE, adverse event; QD, once daily; RP2D, recommended Phase II dose; TEAEs, treatment emergent adverse event; w, weeks.
