# Supplementary material for: Camonsertib, an ATRi, in Combination with Low-Dose Gemcitabine in Solid Tumors with DNA Damage Response Aberrations: Preclinical and Phase Ib Results
Source: Clin Cancer Res. 2026 Jan 21;32(8):1411–23. doi: 10.1158/1078-0432.CCR-25-2240 (PMC13080318; doi:10.1158/1078-0432.CCR-25-2240)
Supplement: Supplementary Table S5 — Drug combination pharmacokinetic parameters [file ccr-25-2240_supplementary_table_s5_suppts5.docx]

| **Supplementary Table S5:** Camonsertib pharmacokinetic parameters in camonsertib and gemcitabine combination – Cycle 1 Day 1 | | | | | | | |
| --- | --- | --- | --- | --- | --- | --- | --- |
| Camonsertib dose (mg) |  | C_max_  (µg/mL) | C_max/D_  [(µg/mL)/mg] | AUC_last_  (µg.h/mL) | AUC_last/D_  [(µg.h/mL)/mg] | AUC_0-4_  (µg.h/mL) | AUC_0-4/D_  [(µg.h/mL)/mg] |
| 80 | **N** | 74 | 74 | 74 | 74 | 74 | 74 |
|  | **Mean** | 3.9 | 0.0483 | 22.0 | 0.275 | 8.81 | 0.110 |
|  | **SD** | 2.07 | 0.0259 | 10.1 | 0.126 | 4.52 | 0.0641 |
|  | **CV%** | 53.6 | 53.6 | 45.9 | 45.9 | 51.3 | 51.3 |
| 120 | **N** | 2 | 2 | 2 | 2 | 2 | 2 |
|  | **Mean** | 3.43 | 0.0286 | 24.9 | 0.208 | 7.69 | 0.0641 |
|  | **SD** | 2.38 | 0.0198 | 8.78 | 0.0732 | 5.64 | 0.0470 |
|  | **CV%** | 69.3 | 69.3 | 35.3 | 35.3 | 73.4 | 73.4 |

Note: Data cut-off date of December 11, 2024.

AUC_0-last_, area under the curve from time 0 to last quantifiable concentration; AUC_0-last/D_, AUC_0-last_ after dose; AUC_0-4/D_, AUC from 0 h to 4 h after dose; C_max/D_, maximum plasma concentration after dose; CV, coefficient of variation; D, day; h, hour; SD, standard deviation.
