# Supplementary material for: Camonsertib, an ATRi, in Combination with Low-Dose Gemcitabine in Solid Tumors with DNA Damage Response Aberrations: Preclinical and Phase Ib Results
Source: Clin Cancer Res. 2026 Jan 21;32(8):1411–23. doi: 10.1158/1078-0432.CCR-25-2240 (PMC13080318; doi:10.1158/1078-0432.CCR-25-2240)
Supplement: Supplementary Table S6 — Additional efficacy parameters [file ccr-25-2240_supplementary_table_s6_suppts6.docx]

| **Supplementary Table S6.** Summary of efficacy parameters by arm, dose, and tumor type | | | | |
| --- | --- | --- | --- | --- |
| Efficacy parameter | **Arm 1**  Gemcitabine  1000–400 mg/m^2^ | **Arm 2**  Gemcitabine  100–200 mg/m^2^ | **Proposed expansion dose**  Camonsertib 80mg QD/  Gemcitabine 400mg, 3/4d, 1/1w | **All doses/schedules** |
| **Overall response rate^a^,** *n/N* (%) | | | | |
| All tumor types | 5/42 (11.9) | 3/25 (12.0) | 2/25 (8.0) | 8/67 (11.9) |
| Non-gynecological | 0/13 | 1/14 (7.1) | 0/1 | 1/27 (3.7) |
| Gynecological | 5/29 (17.2) | 2/11 (18.2) | 2/24 (8.3) | 7/40 (17.5) |
| Ovarian | 4/26 (15.4) | 2/10 (20.0) | 2/23 (8.7) | 6/36 (16.7) |
| **RECIST response rate,** *n/N* (%) | | | | |
| All tumor types | 5/41 (12.2) | 2/25 (8.0) | 2/25 (8.0) | 7/66 (10.6) |
| Non-gynecological | 0/12 | 1/14 (7.1) | 0/1 | 1/26 (3.8) |
| Gynecological | 5/29 (17.2) | 1/11 (9.1) | 2/24 (8.3) | 6/40 (15.0) |
| Ovarian | 4/26 (15.4) | 1/10 (10.0) | 2/23 (8.7) | 5/36 (13.9) |
| **Clinical benefit rate^b^,** *n/N* (%) | | | | |
| All tumor types | 19/42 (45.2) | 11/25 (44.0) | 11/25 (44.0) | 30/67 (44.8) |
| Non-gynecological | 3/13 (23.1) | 7/14 (50.0) | 0/1 | 10/27 (37.0) |
| Gynecological | 16/29 (55.2) | 4/11 (36.4) | 11/24 (45.8) | 20/40 (50.0) |
| Ovarian | 13/26 (50.0) | 4/10 (40.0) | 10/23 (43.5) | 17/36 (47.2) |
| **mPFS**, *weeks* |  |  |  |  |
| All tumor types | 22.4 | 18.0 | 23.3 | 19.0 |
| Non-gynecological | 17.4 | 18.0 | 5.1^c^ | 17.4 |
| Gynecological | 32.6 | 18.0 | 25.4 | 32.6 |
| Ovarian | 25.4 | 18.0 | 23.3 | 25.4 |

Note: Data cut-off date of December 11, 2024.

^a^Overall response rate was defined as RECISTv1.1 or TMR response (CA-125 per GCIG or PSA per PCWG3).

^b^Clinical benefit rate was defined as RECIST or TMR response, or treatment duration of at least 16 weeks without evidence of progression.

^c^n=1

1/1w, 1 week on/1 week off; 3/4d, 3 days on/4 days off; mPFS, median progression-free survival; QD, once daily; RECIST, Response Evaluation Criteria in Solid Tumors v1.1; RP2D, recommended phase II dose; TMR, tumor marker response.
